# A Torque Control Strategy for a Robotic Dolphin Platform Based on Angle of Attack Feedback

**DOI:** 10.3390/biomimetics8030291

**Published:** 2023-07-05

**Authors:** Tianzhu Wang, Junzhi Yu, Di Chen, Yan Meng

**Affiliations:** 1 State Key Laboratory of Management and Control for Complex Systems, Institute of Automation, Chinese Academy of Sciences, Beijing 100190, China; 2 School of Artificial Intelligence, University of Chinese Academy of Sciences, Beijing 100049, China; 3 State Key Laboratory for Turbulence and Complex Systems, Department of Advanced Manufacturing and Robotics, College of Engineering, Peking University, Beijing 100871, China; di.chen@pku.edu.cn (D.C.); mengyan2022@pku.edu.cn (Y.M.)

**Keywords:** robotic dolphin, torque control, angle of attack, motion improvement

## Abstract

Biological fish can always sense the state of water flow and regulate the angle of attack in time, so as to maintain the highest movement efficiency during periodic flapping. The biological adjustment of the caudal fin’s angle of attack (AoA) depends on the contraction/relaxation of the tail muscles, accompanying the variation in tail stiffness. During an interaction with external fluid, it helps to maintain the optimal angle of attack during movement, to improve the propulsion performance. Inspired by this, this paper proposes a tail joint motion control scheme based on AoA feedback for the high-speed swimming of bionic dolphins. Firstly, the kinematic characteristics of the designed robot dolphin are analyzed, and the hardware basis is clarified. Second, aiming at the deficiency of the tail motor, which cannot effectively cooperate with the waist joint motor during high-frequency movement, a compensation model for the friction force and latex skin-restoring force is designed, and a joint angle control algorithm based on fuzzy inference is proposed to realize the tracking of the desired joint angle for the tail joint in torque mode. In addition, a tail joint closed-loop control scheme based on angle of attack feedback is proposed to improve the motion performance. Finally, experiments verify the effectiveness of the proposed motion control scheme.

## 1. Introduction

Compared with robotic dolphins, biological dolphins have unique advantages in terms of motion. After parameter optimization, robotic dolphins often achieve excellent performance at certain speeds, but it is difficult to maintain excellent performance over a wide range. Considering the propulsion method of cetaceans, the fluke is a key organ for dolphin movement and the most important component in maintaining balance and controlling the speed in water. Depending on the rapid contraction and relaxation of muscles, coupled with a strong tail stock, biological dolphins drive a third of their bodies, producing powerful propulsion. Research has shown that fish actively contract their muscles to adjust the stiffness of their tails during swimming. For example, Flammang et al. [1] found that the bluegill sunfish (*Lepomis macrochirus*) stiffened its tail as its speed increased. Long et al. [2] also showed that the American eel (*Anguilla rostrat*) could increase its body stiffness by contracting its muscles and obtain net thrust during steady state swimming. Despite the need for further study on the mechanisms by which biological fish optimize their movement by adjusting the stiffness of their propulsion organs, this phenomenon serves as inspiration for bionic robotic dolphin research.

To optimize the performance of underwater bionic robots, researchers have attempted to introduce stiffness modulation mechanisms. For example, Chen et al. [3] and Tong et al. [4] installed passive stiffness components (torsion springs) in the tails of their robotic fish, and other researchers used adjustable stiffness devices [5,6], all of which improved the performance of the robots. In addition, some researchers have used special driving mechanisms to achieve variable stiffness effects, such as Park et al. [7], who used artificial tendons to simulate the stiffness of fins. Zhong et al. [8] showed that the optimal stiffness must change with the swimming speed to maintain high efficiency. If bionic robots wish to improve their performance over a wide speed range by changing their stiffness, they must calibrate the optimal parameters in advance and adjust the stiffness of the driving mechanism according to the motion state. This method has limited applicability.

The angle of attack (AoA), which refers to the angle between the relative water flow and the chord of a fin-like structure, is believed to play a crucial role in propulsion based on an oscillating hydrofoil [9,10,11]. In steady fluid, the lift of a hydrofoil increases faster than its drag with an increasing AoA up to a critical value. However, if the critical AoA is exceeded, the drag increases continuously while the lift decreases sharply, which is known as the stall condition. Dynamic stall can delay the static stall and increase the critical AoA in the case of an oscillating hydrofoil [12]. Thus, with the assistance of flow sensing, robotic fish can adjust their AoA accordingly to maintain maximum net thrust over a periodic stroke. Shen et al. investigated the wing motion of a real penguin and found that the feathering motion enabled active control of the AoA to improve the swimming performance [13]. Toward the attitude holding task of a robotic fish swimming in the real world, Zheng et al. proposed a deep reinforcement learning (DRL)-based policy to maintain the desired angle of attack [14]. Studies on cetaceans also suggest that a high AoA may help them to obtain thrust from water [15,16,17]. Furthermore, numerical simulations of a fish-like swimmer demonstrated that a leading-edge vortex is formed as the AoA increases [18]. The vortex is widely regarded as being correlated with propulsion, acceleration, and efficiency [19,20,21]. Therefore, based on these findings, it is reasonable to conclude that the AoA has great potential value in studying propulsion or efficiency for bio-robotics. Specifically, biological fluke angle adjustment relies on the contraction/relaxation of the tail muscles, and with changes in tail “stiffness”, it can be used to find the optimal attack angle during movement in interaction with an external fluid.

Inspired by this phenomenon, the motivation of this study is as follows: when the caudal joint undergoes stable motion with a set of fixed parameters (such as amplitude and frequency), the optimal joint angle for the caudal joint is sought by incorporating feedback data on the attack angle, aiming to achieve a higher speed or energy efficiency. The main contributions of this method are twofold. Firstly, the adjustment of the caudal joint angle is performed in real time based on the feedback data of the attack angle, which closely resembles the behavior of biological fish. In contrast, in previous oscillatory motion, multiple experiments are required to find the optimal parameters (amplitude and phase offset). Secondly, this study utilizes the torque mode of the motor to control the caudal joint angle. Compared to the position mode, under equivalent conditions, the caudal fin exhibits smoother performance. Theoretically, this method can adapt to higher upper limits of frequency.

This paper proposes a caudal joint motion control scheme based on attack angle feedback for the high-speed swimming of bionic robotic dolphins, aiming to provide a reference for relevant research. Firstly, the kinematic characteristics of the designed bionic robotic dolphin are analyzed, elucidating the hardware basis of bionic robotic dolphins. Secondly, to solve the problem whereby the caudal motor cannot effectively cooperate with the waist joint motor during high-frequency motion, a friction force and latex skin recovery force compensation model is designed, and a joint angle control strategy based on fuzzy inference is proposed to achieve the expected joint angle tracking of the caudal joint in torque mode. Finally, to address the problem of the limited optimal flapping frequency range for open-loop control laws, a closed-loop control scheme based on the attack angle feedback of the caudal joint is proposed to improve the motion performance. Finally, a pool experiment is conducted to verify the effectiveness of the proposed motion optimization scheme.

The remainder of this paper is organized as follows. Section 2 introduces the motion characteristics of the robotic dolphin’s joint. Section 3 introduces the optimal AoA control law. The control algorithm of the caudal joint in torque mode is proposed in Section 4. The control strategy of the caudal joint based on AoA feedback is presented in Section 5. Section 6 presents the experiments, and Section 7 provides the conclusions of this paper.

## 2. Analysis of the Motion Characteristics of Robotic Dolphin

### 2.1. Overview of the Robotic Dolphin

**Figure 1:**
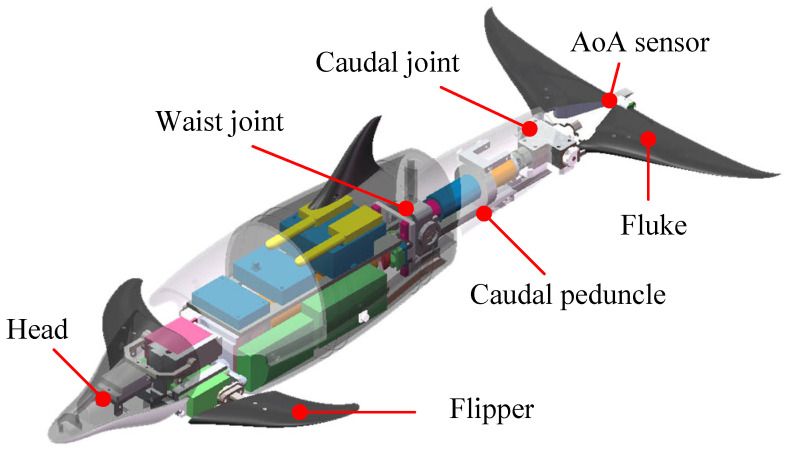
Mechanical design of the robotic dolphin.

Figure 1 illustrates the schematic diagram of the robotic dolphin. The biomimetic design of the robotic dolphin is based on the spotted dolphin (*Stenella attenuata*), using its streamlined body shape to minimize hydrodynamic resistance during underwater locomotion. To emulate the typical dorso-ventral swimming pattern of dolphins, the propulsion system of the robotic dolphin is equipped with two high-performance brushless DC motors (MAXON EC4-pole 200 W and 90 W) as power sources for the waist joint and caudal joint, ensuring strong thrust. The fluke, connected to the caudal joint motor by a bevel gear box, serves as the main source of forward thrust. At the trailing edge of the fluke, an AoA sensor designed by our team [22] is installed to provide real-time AoA data feedback on the fluke during motion.

In terms of the electronic system, various components, including sensors, an embedded controller chip, communication modules, and batteries, are housed within the body of the robotic dolphin to enable untethered autonomous swimming. Specifically, a miniature attitude heading reference system (AHRS, MicroStrain, 3DM-GX5-25) is installed in the head region to provide attitude data for the robotic dolphin. Additionally, dedicated motor drivers (MAXON, EPOS4 50/8 and 50/5) are employed to obtain the state information of the joints, such as their speed and position. Control commands are wirelessly transmitted to the dolphin’s internal system via an RF communication module. For the detailed specifications of the robotic dolphin, please refer to Table 1.

### 2.2. Mechanism Analysis

The caudal peduncle of the robotic dolphin is primarily driven by the waist motor, which is responsible for driving all the components on the caudal peduncle. From a mechanical perspective, the waist joint serves as a forceful lever, thus bearing the largest load. To achieve the reciprocating up-and-down flapping of the fluke, a reversing scheme utilizing a eccentric rotor and a linear guide in combination has been designed for the waist joint.

**Figure 2:**
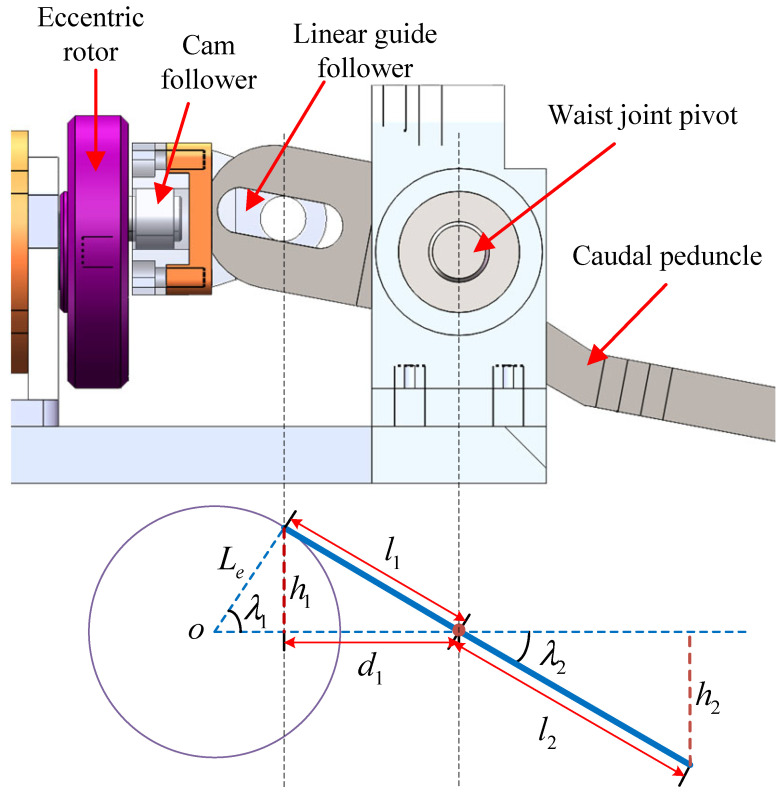
Schematic diagram of the waist joint.

Figure 2 illustrates the schematic diagram of the waist joint. The motor operates in profile velocity mode (PVM) to ensure the stability of the motor output. Meanwhile, the control law of caudal joint $\lambda_{2}$ is determined by the following equation:

(1) $$\left\{\begin{array}{c} \lambda_{1}=\omega t\\ \lambda_{2}=\mathrm{arc}\tan\frac{h_{1}}{d_{1}} \end{array}\right.,$$

where $\lambda_{1}$ represents the rotational angle of the eccentric rotor, and ω represents the angular velocity.

Point *O* in Figure 2 represents the center of the eccentric rotor, which corresponds to the waist motor shaft. The horizontal extension line from *O* coincides with the extension line of the motor shaft and marks the boundary between the up-and-down flapping of the caudal peduncle. The rotational axis of the waist joint also coincides with the motor output shaft. $h_{1}$ represents the vertical distance from the cam follower to the axis of the motor output shaft. The linear guide follower moves up and down and touches the caudal peduncle. $l_{1}$ represents the distance from the contact point to the axis of the waist joint. The caudal peduncle is designed with a slot at the end to match the movement of the cam follower, which means that $l_{1}$ varies with movement. $d_{1}$ represents the horizontal distance from the cam follower to the axis of the waist joint. According to trigonometric functions, we have

(2) $$\left\{\begin{array}{c} h_{1}=L_{e}\sin\lambda_{1}\\ h_{2}=l_{2}\sin\lambda_{2} \end{array}\right.,$$

where $L_{e}$ represents the distance from cam follower to the center of the eccentric rotor, $l_{2}$ represents the distance from the fluke to the waist motor shaft, and $h_{2}$ represents the vertical distance from the fluke to the axis of the caudal motor shaft, representing the lateral displacement distance of the end of the caudal peduncle.

Combining Equations (1) and (2), we have

(3) $$h_{2}=l_{2}\sin\left(\mathrm{arc}\tan\frac{l_{e}\sin\left(\omega t\right)}{d_{1}}\right).$$

For the robotic dolphin utilized in this study, there is $d_{1}=23$ mm, $L_{e}=8.6$ mm, and $l_{2}=148$ mm, and the amplitude of the waist angle is calculated to be 20.5${}^{\circ }$. In addition, the maximum amplitude of the waist joint is determined by $L_{e}$ and can be adjusted. The maximum vertical displacement distance of the caudal peduncle edge is 51.8 mm, which is only 35% of the length of the caudal peduncle. As the propulsion force mainly comes from the fluke rather than the caudal peduncle, the caudal peduncle does not need to have a large swing.

**Figure 3:**
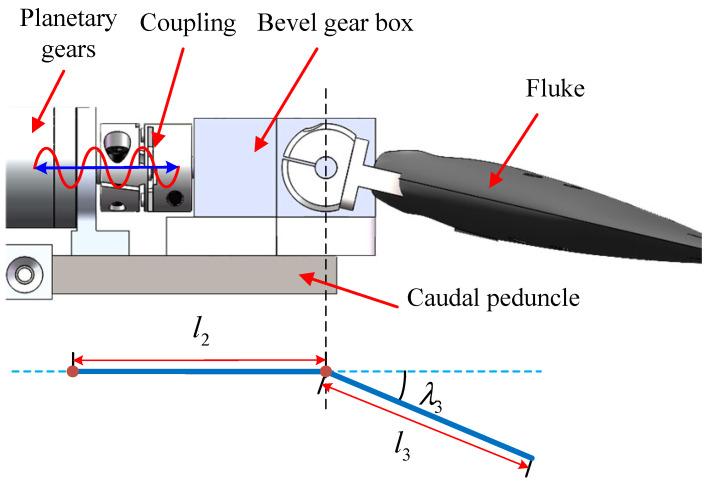
Schematic diagram of the caudal structure.

Figure 3 illustrates the schematic diagram of the caudal structure. The fluke is driven through a 1:1 bevel gearbox by the caudal motor. The caudal joint angle $\lambda_{3}$ is obtained by position encoder feedback. This caudal joint has two characteristics: firstly, the fluke flaps up and down, and both the upper and lower position limits are 70${}^{\circ }$; secondly, restricted by the mechanism, the fluke reverses at the amplitude extreme, meaning that the caudal motor has to change the motion direction in each cycle.

### 2.3. Hydrodynamics Analysis

Hydrodynamics is an important source of propulsion for the fluke, and the hydrodynamic characteristics of the fluke directly affect the swimming performance. In this study, the lift and drag model is adopted to analyze the hydrodynamic characteristics of the fluke. Generally speaking, the lift and drag coefficients depend on the morphology of the fluke, and CFD is an effective method of obtaining the hydrodynamic characteristics of irregularly shaped objects [23,24,25].

To reduce the influence of eddy currents on the simulation results, the distance from the velocity inlet to the pressure outlet is greater than three times length of the fluke. A non-structured method is adopted for mesh division. Meanwhile, a five-layer tetrahedral boundary layer mesh is created on the surface of the fluke to refine the boundaries between the experimental object and the environment, to obtain better convergence and analysis results. The fluid is assumed to be incompressible and stable pure water in the simulation. Thus, the low Reynolds number k−ω shear stress transport (SST) turbulence model is selected to solve the equations.

In the CFD simulation, $\alpha \in \left[-\pi /2,\pi /2\right]$ is selected as the experimental variable to solve the lift and drag coefficients of the fluke. Equations for the fitting curves are given as follows:

(4) $$\left\{\begin{array}{cc} C_{l}= & 0.5994\sin\left(1.875\alpha \right)+0.1945\sin\left(3.75\alpha \right)\;\\ \; & +0.1234\sin\left(5.625\alpha \right)+0.0634\sin\left(7.5\alpha \right)\;\\ C_{d}= & 0.4533\sin\left(2.101\alpha \right)-0.3763\sin\left(4.202\alpha \right)\;\\ \; & -0.0219\sin\left(6.303\alpha \right)-0.0213\sin\left(8.404\alpha \right) \end{array}.\right.$$

## 3. Optimal Angle of Attack Control Law

**Figure 4:**
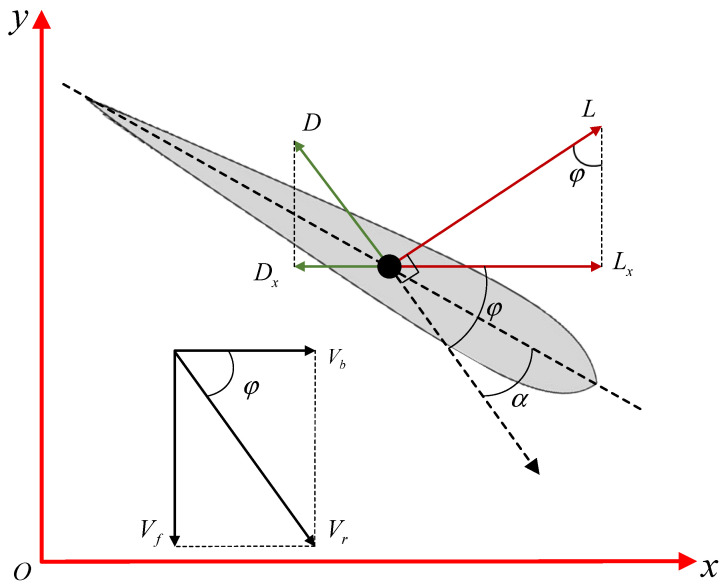
Schematic diagram of the relationship between AoA and the force acting on it.

The angle of attack is the angle between the plane of the fluke and the direction of water flow, which is a key variable in determining the propulsive force generated by the fluke. Figure 4 shows the relationship between the AoA and the forces acting on the fluke. The fluid forces acting on the fluke in water can be divided into two components: lift *L* perpendicular to the direction of motion and drag *D* parallel to the direction of motion. Here, the direction of motion is the absolute velocity $V_{r}$ of the fluke in the real world, which is composed of the forward velocity component $V_{b}$ and the lateral velocity component $V_{f}$. Ignoring surface friction, pressure drag, and gravity, according to the lift–drag model, the force acting on the fluke can be represented as

(5) $$\left\{\begin{array}{c} L_{x}=L\sin\varphi =0.5\rho SC_{l}{V_{r}}^{2}\sin\varphi \\ D_{x}=D\cos\varphi =0.5\rho SC_{d}{V_{r}}^{2}\cos\varphi \end{array}.\right.$$

where $L_{x}$ and $D_{x}$ are the components of lift and drag along the *x*-axis, φ is the angle between the direction of movement of the fluke and the forward direction, ρ is the fluid density, *S* is the area of the fluke under force, and $C_{l}$ and $C_{d}$ are the lift and drag coefficients, respectively, which are related to the shape of the fluke and the AoA. For a given fluke, $C_{l}$ and $C_{d}$ are functions of the AoA, denoted as $C_{l}\left(\alpha \right)$ and $C_{d}\left(\alpha \right)$. The net thrust acting on the fluke in the forward direction can be calculated as

(6) $$T=L_{x}-D_{x}=0.5\rho S{V_{f}}^{2}\left(C_{l}\left(\alpha \right)\sin\varphi -C_{d}\left(\alpha \right)\cos\varphi \right).$$

**Figure 5:**
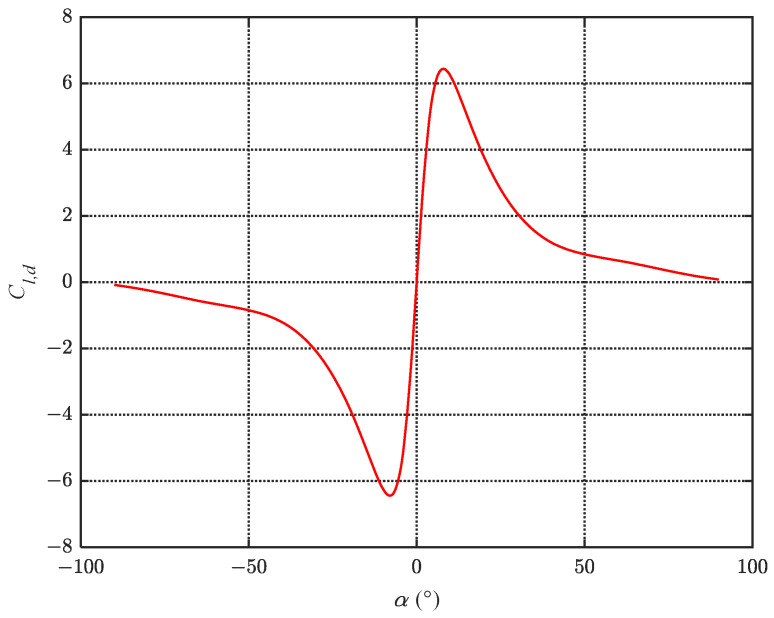
Relationship between AoA and lift-to-drag ratio for the fluke.

When $C_{l}\left(\alpha \right)\sin\varphi -C_{d}\left(\alpha \right)\cos\varphi >0$, the dolphin can obtain net thrust, i.e.,

(7) $$\tan\left(\varphi \right)>\frac{C_{d}\left(\alpha \right)}{C_{l}\left(\alpha \right)},$$

where $C_{l,d}=C_{l}/C_{d}$ is the lift-to-drag ratio of the fluke. Referring to the lift and drag coefficients obtained from the CFD simulation in Section 2.3, the curve of $C_{l,d}$ is as shown in Figure 5. According to Equation (4), the maximum lift coefficient of the fluke is achieved at $\alpha =22.3^{\circ }$, but the maximum lift-to-drag ratio of 6.44 occurs at $\alpha =7.4^{\circ }$. Substituting these data into Equation (7), we obtain

(8) $$\tan\varphi >\frac{1}{6.43}.$$

At this point, $\varphi >8.0^{\circ }$. This means that when the angle between the direction of motion of the fluke and the forward direction is less than $8.0^{\circ }$, the net thrust is negative. In this case, the AoA should be set to zero; otherwise, negative net thrust will be introduced.

The core ideas of fluke AoA control are twofold: to maintain positive net thrust and to keep the fluke AoA as close as possible to the maximum lift coefficient. For the fluke chosen in this study, when the first criterion is met, $\varphi >8.0^{\circ }$ is required. When the second criterion is met, $\alpha =22.3^{\circ }$ is required, where the lift-to-drag ratio is 3.31, which corresponds to $\varphi >16.8^{\circ }$. Therefore, the following law for AoA variation is formulated:

1. When $16.8^{\circ }\leq \varphi$, the attack angle maintains the maximum lift coefficient, i.e., $\alpha =22.3^{\circ }$.
2. When $8.0^{\circ }\leq \varphi <16.8^{\circ }$, the attack angle cannot maintain the optimal lift coefficient; otherwise, it will result in negative net thrust. Therefore, a smoothed curve for α is set in this interval.
3. When $0^{\circ }\leq \varphi <8.0^{\circ }$, $\alpha =0^{\circ }$.

**Figure 6:**
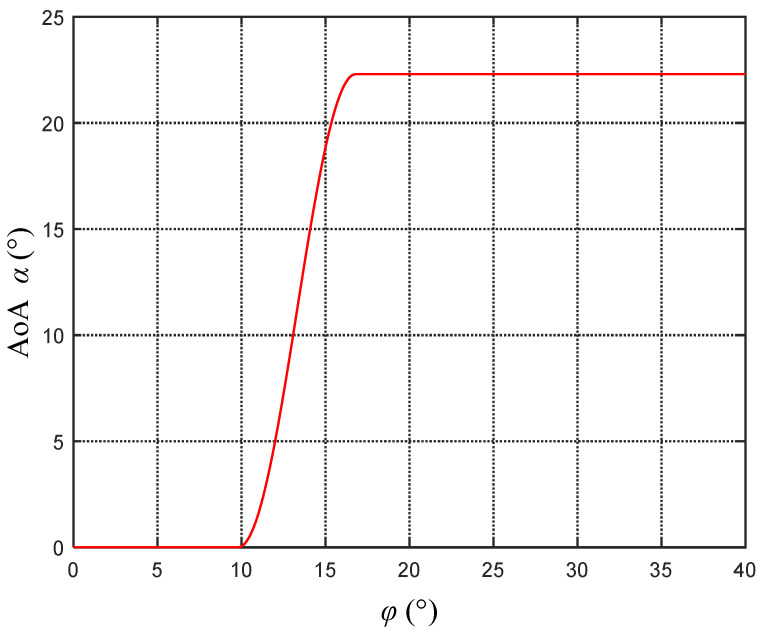
Optimal AoA control law.

Subject to the aforementioned constraints, a smooth variation in the AoA within the interval $\left[8.0^{\circ },16.8^{\circ }\right]$ is ensured by

(9) $$\alpha =\left\{\begin{array}{c} \alpha_{\max},\varphi \in \left[\varphi_{2},90^{\circ }\right]\\ 0.5\alpha_{\max}\left(\sin\left(\left(\varphi -\varphi_{1}\right)\pi /\left(\varphi_{2}-\varphi_{1}\right)-0.5\pi \right)+1\right),\varphi \in \left[\varphi_{1},\varphi_{2}\right)\\ 0^{\circ },\varphi \in \left[0,\varphi_{1}\right) \end{array},\right.$$

where $\varphi_{1}=8.0^{\circ }$, $\varphi_{2}=16.8^{\circ }$, and $\alpha_{\max}=22.3^{\circ }$. For ease of understanding, Equation (9) will be referred to as the optimal AoA control law model in this section. The relationship between the AoA and φ is illustrated in Figure 6.

## 4. Caudal Joint Control in Torque Mode

For multi-joint underwater bionic robots, the undulation frequency, joint angle amplitude, and phase difference are key parameters determining motion performance. Generally speaking, the undulation frequency usually has the most significant effect on the swimming speed, and a considerable proportion of high-speed bionic robots are capable of high-frequency swimming [26,27,28]. However, there is always an upper limit to the undulation frequency when the mechanism characteristics are considered. This paper proposes a joint angle control algorithm based on motor torque mode, which further improves the motion performance of the robotic dolphin compared to the common motor position mode without requiring hardware changes. The proposed method can be widely employed in the joint angle control of bionic robots driven primarily by DC motors.

### 4.1. Problem Description

**Figure 7:**
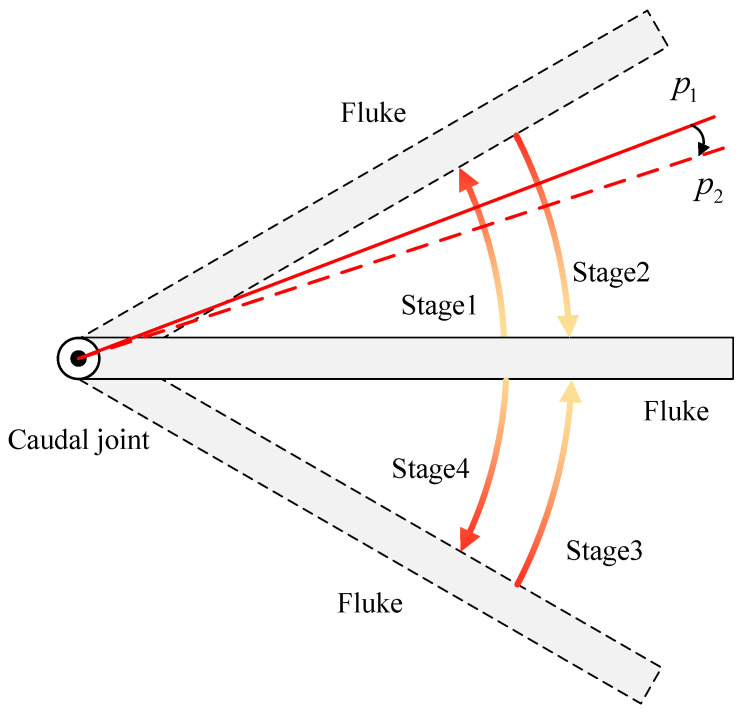
Diagram illustrating the motion of the caudal joint under CST mode.

Considering the actuation frequency limitation of commonly used direct current (DC) motor profile position (PPM) mode, we choose to control the torque of the tail motor and use the cycle synchronous torque mode (CST) of the motor driver to realize joint angle control. A schematic diagram of the fluke movement under CST mode is presented in Figure 7. In this mode, the swinging motion of the fluke within a single cycle is also divided into two stroke phases (stage 2 and 3) and two return phases (stage 1 and 4). In the stroke phase, as the caudal joint angle increases, the output torque of the motor also increases. The reverse process takes place during the return phase, where the motor torque gradually decreases. The above process is similar to adding an adjustable-stiffness torsion spring at the caudal joint, with the stroke and return phases being similar to the processes of energy release and storage of the torsion spring. However, fundamentally, both processes consume energy from the motor, and the motor can adjust the energy storage at any time as needed.

Taking the motion from point $p_{1}$ to point $p_{2}$ as an example, at the beginning of a control cycle, the controller determines the torque within this control cycle based on the current error and the current state of the motor. If the load exceeds the limit during this cycle, it will only increase the position error without damaging the joint. Compared with PPM, the drawback of CST mode is limited control accuracy, while its advantage is that the fluke is approximately a passive joint and can be combined with higher-frequency waist joint flapping under the same conditions.

The extreme performance of CST mode depends on the motor’s heat dissipation, which can be calculated using the Joule integral formula (*I*${}^{2}$*t*):

(10) $$\vartheta =P_{V}\cdot R_{th}\cdot \left\{1-e^{-\frac{t}{\tau_{th}}}\right\}+\vartheta_{a}\cdot e^{-\frac{t}{\tau_{th}}},$$

where ϑ is the actual winding temperature, $P_{V}$ is the heat dissipation loss, $R_{th}$ is the winding resistance value, $\vartheta_{a}$ is the initial measured temperature, and $\tau_{th}$ is the winding thermal time constant.

**Figure 8:**
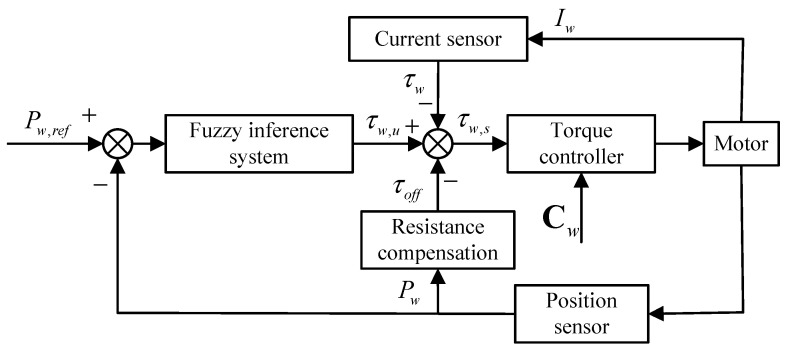
Framework diagram of control algorithm for caudal joint in torque mode.

In brief, the control algorithm for the caudal joint in torque mode is illustrated in Figure 8. Firstly, the deviation between the desired value $P_{w,ref}$ and the current position $P_{w}$ returned by the motor position sensor is obtained. Secondly, a fuzzy inference system is designed to map the current position deviation to the underlying motion parameter $\tau_{w,u}$. Then, the current position $P_{w}$ is input into a friction compensation model to calculate the friction force and the restoring force of the latex skin, and then output the compensating torque $\tau_{off}$. Next, the desired torque $\tau_{w,u}$, compensating torque $\tau_{off}$, and current actual torque $\tau_{w}$ are combined to form the actual torque setpoint $\tau_{w,s}$. Finally, the torque controller integrated within the motor drive converts $\tau_{w,s}$ and other motor parameters $C_{w}$ into control signals for the caudal joint motor. It is worth noting that the “torque controller” depicted in Figure 8 is provided by the motor manufacturer and is embedded within the motor drive board, ensuring that the motor outputs the desired torque according to the given command. With the above controller, the fluke continuously moves along the desired trajectory and eventually flaps in coordination with the waist joint to achieve forward swimming.

### 4.2. Resistance Compensation Model

The basic requirement in torque mode is to achieve precise force transmission between the fluke and the tail motor. In order to improve the accuracy of force transmission, mechanical structure improvements, including the use of industrial cone gearboxes instead of custom-made bevel gears, have been adopted. Another factor affecting the force transmission of the fluke is the joint frictional force and the restoring force of the waterproof latex skin.

**Figure 9:**
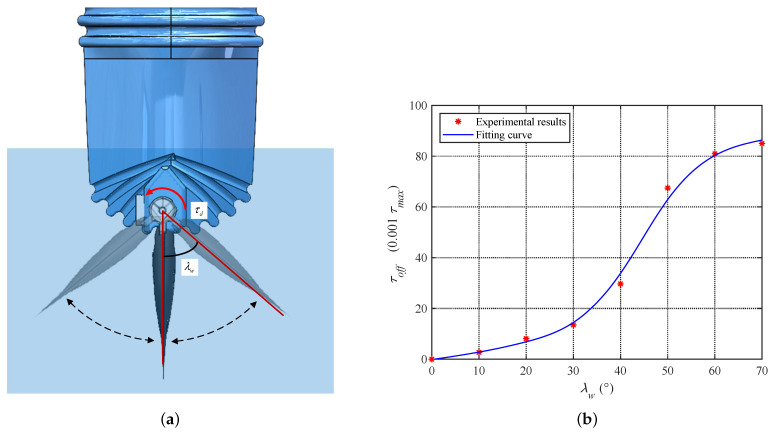
Illustration of resistance on the caudal joint. (**a**) Resistance of waterproof skin. (**b**) Resistance compensation model.

Figure 9a shows a schematic diagram of the waterproof latex skin at the tail end. The waterproof skin is designed with multiple creases at the tail end to provide space for the tail joint’s motion. When the creases are stretched or compressed, there is a restoring force that returns them to their original shape. The magnitude of this restoring force is positively correlated with the deflection angle of the fluke. In addition, there is friction introduced into the movement due to the planetary reducer, coupling, and bevel gearbox connecting the fluke and the motor output shaft. The factors affecting the joint frictional force are numerous and difficult to model accurately. This paper collectively refers to the two types of forces mentioned above as resistance. From the experimental results, it can be seen that the restoring force of the waterproof skin mainly contributes to the resistance, while the joint frictional force accounts for a small proportion.

The resistance of the caudal joint has a significant impact on the force transmission of the fluke, so compensation for this resistance must be included in the control model. Considering that the factors contributing to resistance are rather complex, this paper constructs a resistance compensation model based on experimental data. Specifically, the fluke is balanced to zero buoyancy and immersed in water as shown in Figure 9a. The required torque value for each caudal joint angle is recorded. Figure 9b shows the relationship between the caudal joint angle and the resistance compensation value $\tau_{off}$, where the unit of the compensation value is one thousandth of the rated torque. The equation of the fitted curve for these two variables is

(11) $$\tau_{off}=78.82\sin\left(0.02\lambda_{w}-0.22\right)+15.3\sin\left(0.097\lambda_{w}+1.81\right).$$

### 4.3. Fuzzy Inference System Design

**Figure 10:**
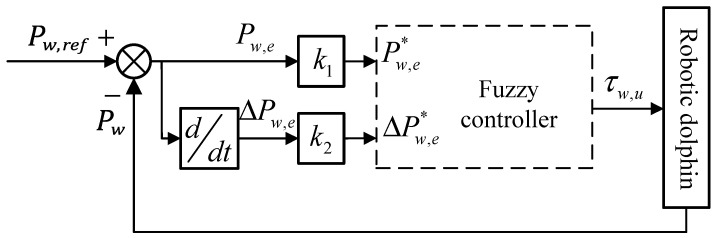
Structure diagram of fuzzy inference system for the caudal joint.

During the flapping process of the fluke, the hydrodynamic forces acting upon it constantly change. The relationship between the output torque of the caudal joint and the actual deflection angle of the fluke is directly affected by the hydrodynamic forces within a control cycle, making it difficult to establish an exact relationship between the two. Therefore, this section adopts a fuzzy inference system to realize the underlying mapping between the position error and expected torque. Figure 10 shows the structure diagram of the fuzzy inference system.

The main purpose of the fuzzy inference system is to generate the control torque of the caudal joint based on the position error of the caudal joint angle. This paper adopts a dual-input single-output fuzzy mapping structure. As shown in Figure 10, the dual input variables are the position error $P_{w,e}$ and the rate of change of position error $\Delta P_{w,e}$, while the single output is the desired torque $\tau_{w,u}$. $P_{w,ref}$ represents the desired position, and $P_{w}$ represents the current actual position returned by the motor driver. Here, $P_{w,ref}\left(k\right)$ and $P_{w}\left(k\right)$, respectively, denote the discrete values at time t(k). The position error $P_{w,e}$ at time t(k) and its rate of change $\Delta P_{w,e}$ are calculated as follows:

(12) $$\left\{\begin{array}{c} P_{w,e}\left(k\right)=P_{w,ref}\left(k\right)-P_{w}\left(k\right)\\ \Delta P_{w,e}\left(k\right)=P_{w,e}\left(k\right)-P_{w,e}\left(k-1\right) \end{array}.\right.$$

In addition, trigonometric and Z-functions are adopted as the membership functions in this paper. $P_{w,e}^{*}$ and $\Delta P_{w,e}^{*}$ represent the fuzzificated $P_{w,e}$ and $\Delta P_{w,e}$, respectively. $\tau_{w,u}$ represents the output of the fuzzy controller, which is used to determine the torque of the caudal joint.

## 5. Control Strategy of Caudal Joint with AoA Feedback

As mentioned earlier, the motors of the robotic dolphin’s waist joint rotate at a constant speed, so the control law for the waist joint is fixed. The motion of the waist joint approximates a sine wave. Therefore, this section focuses on the design of the control law for the caudal joint. The angle of the caudal joint should be actively adjusted according to actual conditions to ensure that the AoA conforms to the optimal AoA control law described earlier.

**Figure 11:**
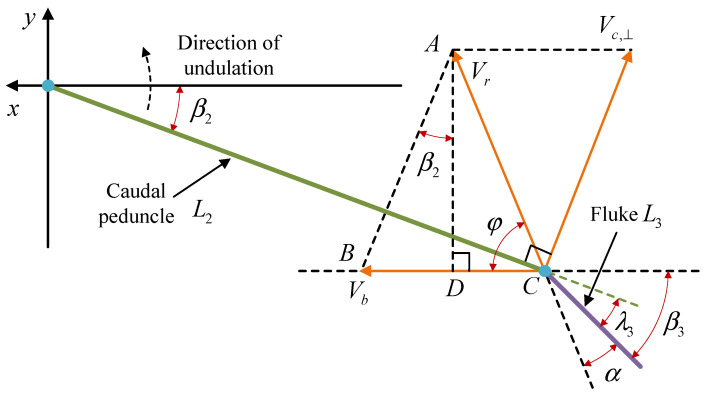
Schematic diagram of the relationship between the caudal peduncle and the fluke under motion.

Figure 11 shows a simplified linkage diagram for the robotic dolphin’s tail stalk and fluke. In this figure, the robotic dolphin moves along the positive *x*-axis, and the tail stalk is flapping upwards. Let $\beta_{2}$ be the angle between the tail stalk and the forward direction, $\beta_{3}$ be the angle between the fluke and the forward direction, $\lambda_{3}$ be the angle of the caudal joint, and $L_{2}$ and $L_{3}$ be the lengths of the simplified tail stalk and fluke linkages, respectively. At this moment, the velocity of the fluke in the world coordinate system is denoted by $V_{r}$, which consists of $V_{b}$, the velocity component of the robotic dolphin along the *x*-axis, and $V_{c,⊥}$, the velocity component of the leading edge of the fluke perpendicular to the tail stalk, which is caused by the rotation of the waist joint. φ represents the angle between $V_{r}$ and $V_{b}$, which can also be viewed as the angle of movement for the fluke and is the main parameter in the optimal AoA control law described earlier. α is the angle between the fluke and $V_{r}$, i.e., the AoA.

To calculate the expected AoA based on the optimal AoA control law, the fluke movement angle φ needs to be calculated first. Examining ▵ABC, we have

(13) $$\varphi =\arctan\frac{\left|AD\right|}{\left|CD\right|}.$$

Based on the geometry, $∠BAD=\beta_{2}$, and we have

(14) $$\left|AD\right|=V_{c,⊥}\cos\beta_{2}.$$

Using the above equation, we can further solve for $\left|CD\right|$:

(15) $$\begin{array}{ccc} \left|CD\right| & = & \left|BC\right|-\left|BD\right|\\ \; & = & V_{b}-V_{c,⊥}\sin\beta_{2} \end{array}.$$

Considering that $V_{c,⊥}$ is the velocity component of the leading edge of the fluke perpendicular to the tail stalk caused by the rotation of the waist joint, its magnitude is

(16) $$V_{c,⊥}={\dot{\beta }}_{2}\cdot L_{2}.$$

Combining Equations (13)–(16), we obtain

(17) $$\varphi =\arctan\frac{{\dot{\beta }}_{2}L_{2}\cos\beta_{2}}{V_{b}-{\dot{\beta }}_{2}L_{2}\sin\beta_{2}}.$$

**Figure 12:**
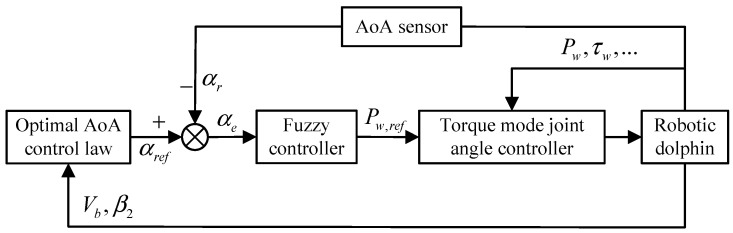
Framework diagram of the closed-loop control algorithm for the caudal joint based on AoA feedback.

Therefore, we establish a closed-loop control algorithm for the caudal joint based on AoA feedback, as shown in Figure 12. Firstly, based on the internal state data of the robotic dolphin, we use the optimal AoA control law to calculate the expected AoA $\alpha_{ref}$ for the current control cycle. Secondly, we use the feedback data from the AoA sensor to calculate the AoA deviation $\alpha_{e}$ and design a fuzzy controller to map $\alpha_{e}$ to the expected angle of the caudal joint. The fuzzy control process is similar to the design approach in Section 4 and will not be repeated here. Finally, we adjust the angle of the caudal joint to the expected value.

The control framework shown in Figure 12 requires real-time feedback on the pitch angle, joint angle, and swimming speed to determine the optimal expected torque. The pitch angle of the robotic dolphin is provided by the AHRS in the head, the joint angle and angular velocity data are from the motor driver, and the determination of the swimming speed requires external measurement or manual intervention.

## 6. Experiments and Analysis

To verify the effectiveness of the proposed torque mode joint angle control algorithm and the closed-loop caudal joint control algorithm based on AoA feedback, we conduct pool experiments. Firstly, the experimental scenario for the bionic robotic dolphin is introduced, followed by a detailed description of the experimental process for the torque mode joint angle control algorithm. Finally, the effectiveness of the AoA feedback control algorithm is verified through comparative experiments.

### 6.1. Pool Experiment Environment Settings

**Figure 13:**
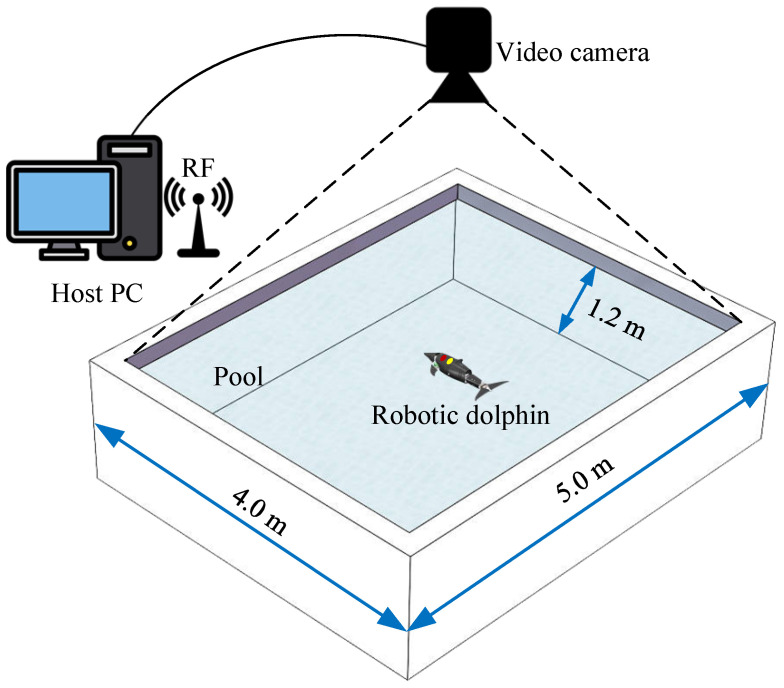
Schematic of the experimental apparatus.

The experimental platform mainly consists of four parts, i.e., a laboratory pool, a global vision measurement system, a host computer, and a robotic dolphin. As illustrated in Figure 13, the pool has a size of 5.00×4.00×1.20 m${}^{3}$. The global vision measurement system mainly consists of a color camera located above the pool with a resolution of 1294×964 pixels. During the experiment, the robotic dolphin’s head is marked with red and yellow adhesive tape, and the global vision measurement system is responsible for collecting and detecting the motion of the robotic dolphin, returning its coordinates in a two-dimensional plane. The host computer is responsible for sending control commands to the robotic dolphin and receiving data from it. The robotic dolphin itself also has a gyroscope and accelerometer for feedback on its attitude and velocity data. In addition, in the experiment to verify the effectiveness of the AoA feedback algorithm, the speed information obtained by the global vision measurement system is sent to the robotic dolphin through the host computer.

### 6.2. Experimental Analysis of Torque Mode Joint Angle Control

Based on the above experimental environment, this section designs three types of experiments—static, anti-interference, and swimming—to verify the torque mode fluke joint angle control algorithm. The aforementioned experiments are conducted using the control scheme depicted in Figure 8. In the experiment, a control command is generated every 20 ms, and the sensor data acquisition is synchronized with this interval. Considering that some modules in the control block are provided by the motor driver manufacturer, it was challenging to collect and compare data from these modules. Therefore, in this section of the experiments, only joint angle data were compared. To quantitatively evaluate the control effect, the average error between the actual joint angle and the expected joint angle is adopted.

#### 6.2.1. Static Experiment

**Figure 14:**
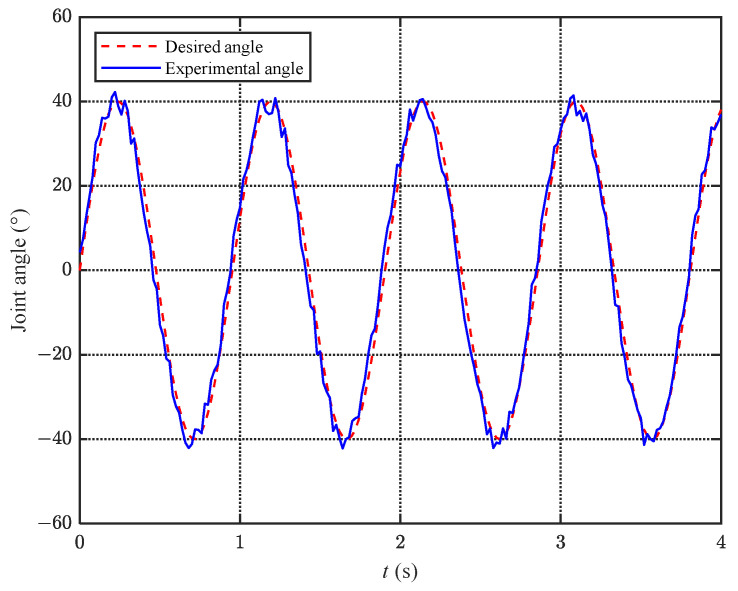
Tracking curve of the fluke joint angle under static experiments.

To verify the effectiveness of the proposed caudal joint control algorithm, a static experiment is conducted, where the bionic robotic dolphin is fixed underwater and the caudal joint tracks a specified waveform, which is a sine wave f(t)=40sin(2πt). The static experiment mainly tests the performance of the control algorithm in a simple scenario. Figure 14 shows the tracking effect of the fluke joint angle in the static experiment. As can be seen from the figure, based on torque control, the caudal joint angle can track the sine wave well, with a calculated error metric $E_{w}=2.53^{\circ }$. It is worth noting that the actual caudal joint angle jumps in a short period of time due to the continuous changes in the hydrodynamic load on the fluke. The torque mode control algorithm does not rely on absolute position feedback, so the average error of $2.53^{\circ }$ meets the control requirements.

#### 6.2.2. Anti-Interference Experiment

**Figure 15:**
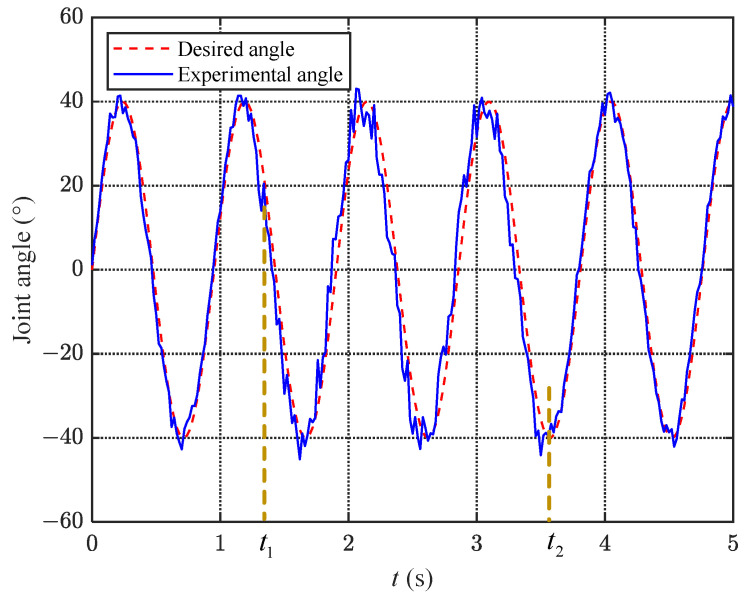
Graph of caudal joint variation under anti-interference experiments.

To verify the robustness of the proposed torque mode joint angle control algorithm, an anti-interference experiment is conducted. Compared to the static experiment, in the anti-interference experiment, the experimenter randomly selected a moment to hold an aluminum alloy rod and made contact with the fluke of the robotic dolphin, without applying a force significantly exceeding the limit of the fluke’s motion. In Figure 15, the fluke is influenced at $t_{1}=1.3$ s by external disturbances until $t_{2}=3.5$ s. As can be seen from the figure, the external disturbance increases the tracking error during the interference time period. After calculation, the average error increases from $E_{w}=2.67^{\circ }$ to $E_{w}=5.21^{\circ }$ with the addition of the disturbance, but it still remains within $6^{\circ }$. With the disturbance removed, after a short period of fluctuation, the average error stabilizes at $E_{w}=2.83^{\circ }$. The anti-interference experiment demonstrates the robustness of the proposed torque mode joint angle control algorithm.

#### 6.2.3. Dynamic Experiment

**Figure 16:**
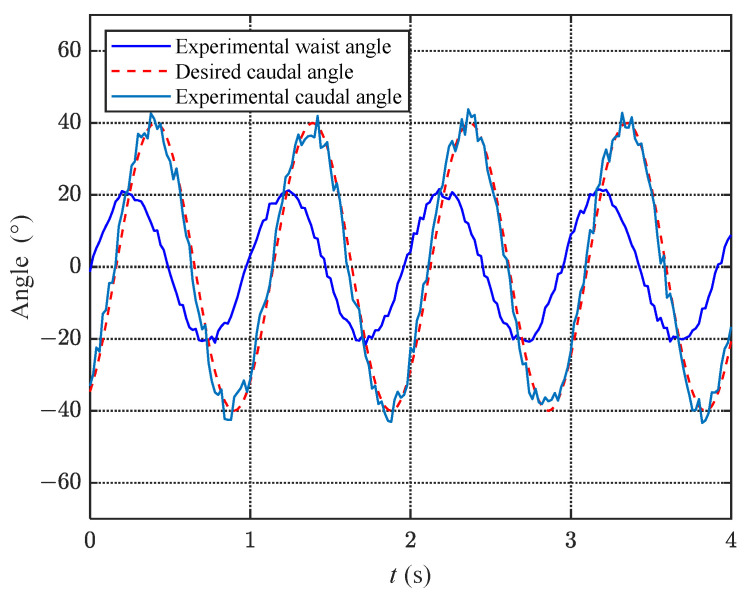
Relationship graph of waist and caudal joint angles under dynamic experiments.

To verify the effectiveness of the proposed torque mode joint angle control algorithm during motion, we conducted dynamic experiments on the bionic robotic dolphin. In the dynamic experiment, the waist motor rotated at a uniform speed and the fluke flapped at a frequency of approximately 1 Hz. The motion law of the waist joint was assumed to be a sine wave, and the fluke moved at a phase difference of $-60^{\circ }$ with an amplitude of 40${}^{\circ }$, which matched the waist joint motion. Figure 16 shows a comparison of the waist and caudal joint angles in the dynamic experiment. As can be seen from the figure, although the waist motor moved uniformly, the curve of the waist joint was not smooth since the designed switching mechanism was a force lever that amplified changes in external loads. After calculation, the average error of the caudal joint angle in the dynamic experiment was $E_{w}=3.82^{\circ }$, which increased compared to the static experiment but still met the control requirements.

### 6.3. Analysis of Angle of Attack Feedback Control Experiment

To verify the effectiveness of the proposed caudal joint motion control algorithm based on angle of attack feedback, this section designs dynamic and power experiments by comparing it with conventional position-based control algorithms.

### 6.4. Tracking Error Analysis

**Figure 17:**
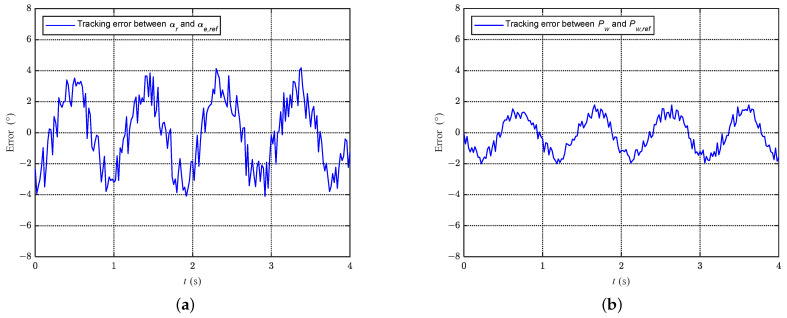
Error analysis. (**a**) Errors between $\alpha_{r}$ and $\alpha_{e,ref}$. (**b**) Errors between $P_{w}$ and $P_{w,ref}$.

Before conducting the aforementioned two experiments, we first analyzed the tracking errors in the AoA feedback experiment. The experimental setup was similar to the dynamic experiment described in the previous section, with the waist joint rotating at a constant speed and the fluke flapping at approximately 1.0 Hz. From Figure 17a, it can be observed that the mean tracking error between $\alpha_{r}$ and $\alpha_{r}ef$ is 1.95${}^{\circ }$. In comparison, Figure 17b shows that the average tracking error between $P_{w}$ and $P_{w,ref}$ is 0.99${}^{\circ }$. There may be two reasons for this phenomenon. Firstly, the AoA for the fluke is obtained from a self-made sensor, while the joint angle is directly acquired from the encoder of the motor, which has better accuracy and stability. Secondly, the AoA is part of the outer loop in the control framework, and, compared to the inner loop variable, the outer loop variable is more prone to accumulating errors.

**Figure 18:**
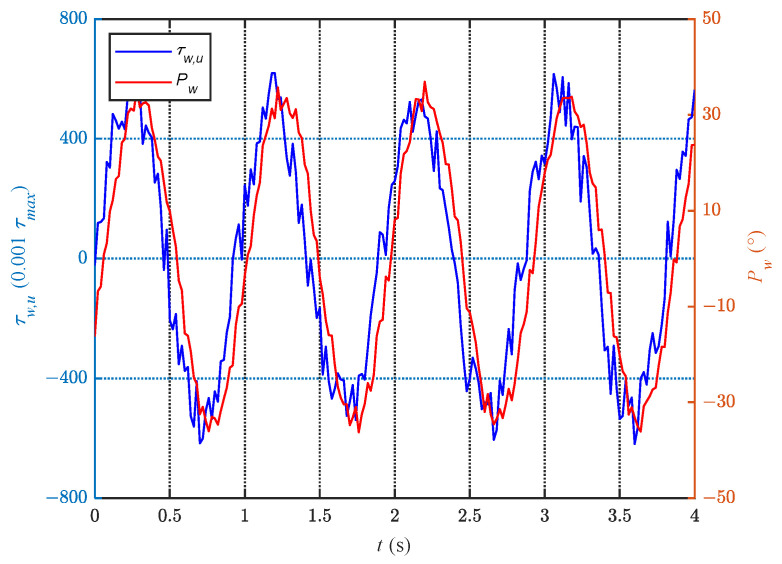
Corresponding relationship between $\tau_{w,u}$ and $P_{w}$.

Meanwhile, Figure 18 illustrates the corresponding relationship between $\tau_{w,u}$ and $P_{w}$. From the Figure 18, it can be observed that the changes in $\tau_{w,u}$ occur earlier in phase than those in $P_{w}$, and $\tau_{w,u}$ exhibits larger variations in errors. The sudden changes in the values of $\tau_{w,u}$ observed in the experiments are possibly related to the torque mode. This mode is prone to large variations in input quantities, leading to abrupt changes.

#### 6.4.1. Dynamic Experiment

The dynamic experiment included a swimming test controlled by the proposed method and a comparison experiment using position-based mode control. Specifically, in the comparative experiment, the waist joint used PVM mode to move uniformly, and the tail motor used PPM mode to control the caudal joint angle by controlling the motor’s forward and reverse rotations. The amplitude of the caudal joint angle in the comparison group was set to $45^{\circ }$, with a phase difference of $-80^{\circ }$ and $-60^{\circ }$ with respect to the waist joint motion for comparison groups 1 and 2, respectively. Except for these differences, the parameters of the proposed method and the comparison group were identical.

**Figure 19:**
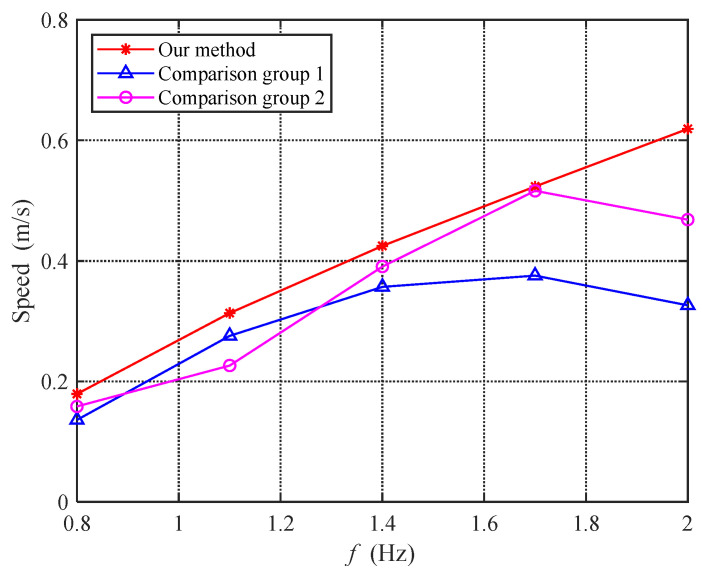
Comparison graph of speed under dynamic experiments.

Figure 19 shows the speed analysis of the above three experiments. As can be seen from the figure, the speed achieved by the proposed method is superior to that of the comparison group at all frequencies. However, the main purpose of this study was not simply to compare swimming speeds, since there must be a set of parameters that perform better for certain frequencies than the proposed method. Looking at the two lines of the comparison group, each performed outstandingly at some frequencies, but, after exceeding the optimal frequency range, the growth in swimming speed was no longer significant. At high frequencies, the comparison group even experienced a decrease in swimming speed. On the other hand, the proposed method achieved a good mapping between the stroke frequency and swimming speed over a wider frequency range, which is difficult to achieve with current motion strategies based on fixed control laws.

#### 6.4.2. Power Experiment

**Figure 20:**
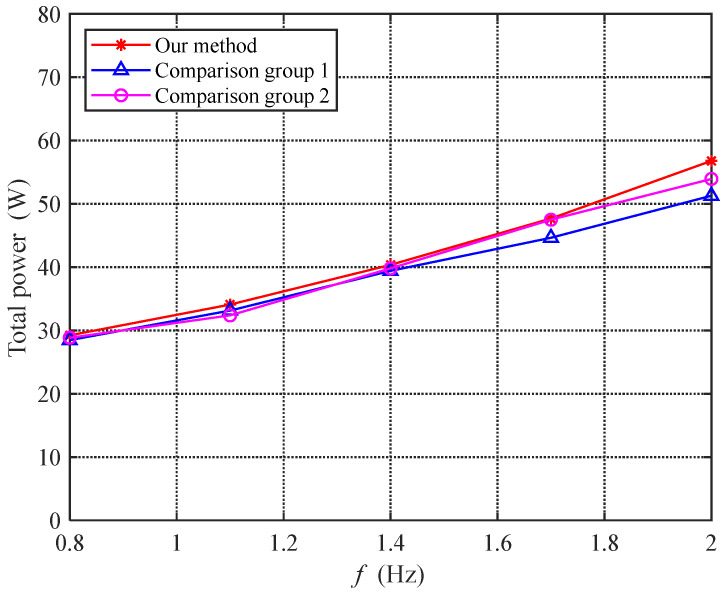
Comparison graph of power under power experiment.

To further analyze the effectiveness of the proposed method, this section tested the swimming power of the bionic robotic dolphin. The acquisition of power depends on the current feedback function of the motor driver. Specifically, the motor driver uses a first-order low-pass filter with a cutoff frequency of 50 Hz to filter high-frequency current data and feed back the current average value to the data collection and processing unit at a maximum sampling frequency of 200 Hz. Then, the data processing unit uses a moving average filtering method to denoise the collected current information and calculates the total power consumption of the bionic dolphin under different experimental conditions. Figure 20 shows the comparison of the total power consumption of the three experiments at different frequencies. As can be seen from the figure, the power consumed by the three experiments is very similar. Therefore, the impact of the proposed method on power consumption is very limited.

Comparing the results in Figure 19 and Figure 20, we can see that the speeds are different even when the total power consumption is the same. Therefore, we introduce the concept of the cost of transport (COT) to measure the propulsion efficiency of the bionic robotic dolphin. COT = P/V, which indicates the ratio of power consumption to speed and is often used to measure the energy consumed by a robot per unit distance traveled. The lower the COT value, the higher the propulsion efficiency.

**Figure 21:**
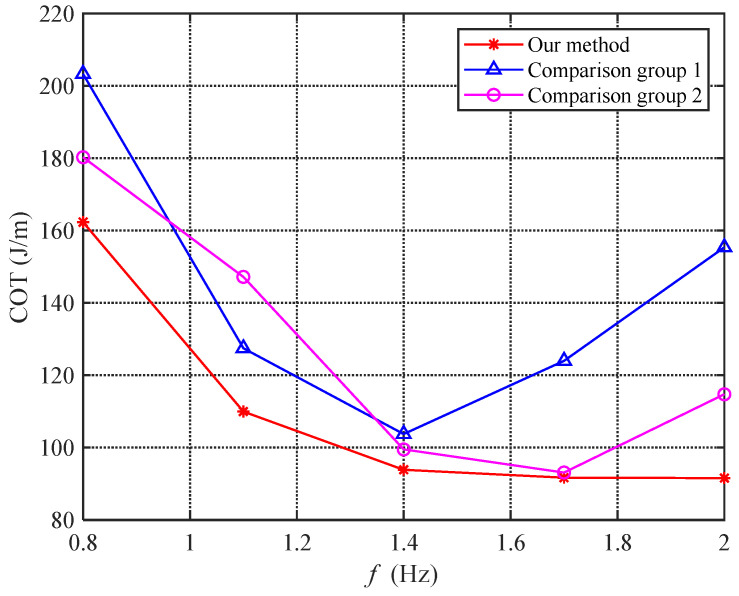
Comparisons of COT under different methods.

Figure 21 shows the COT values of the three experiments at different frequencies. As can be seen from the figure, the proposed method has a significantly lower COT value than the comparison group, indicating that the angle of attack feedback control algorithm improves the propulsion efficiency of the bionic robotic dolphin. The optimal propulsion efficiency of the bionic robotic dolphin is achieved at a frequency of 1.7 Hz, with a value of 91.7 J/m. Calculated based on the battery (29.6 V, 1300 mAh) carried by the bionic robotic dolphin, the maximum cruising range is 1510.7 m.

## 7. Conclusions

In this paper, a bionic motion control strategy based on angle of attack feedback is proposed to pursue the high-speed swimming of bionic robot dolphins. First, the motion characteristics of the designed robot dolphin are analyzed, illustrating the hardware basis of the designed control strategy for a robot dolphin. Second, to solve the problem whereby the motor cannot effectively follow the given trajectory during high-frequency oscillation, a torque mode joint angle control method is proposed. By utilizing the actuation characteristics, the motor can effectively match the high-frequency beating by controlling the motor torque to track the desired angle. Finally, aiming at the problem wherein the optimal beat frequency range of the open-loop control law is limited, a tail joint closed-loop control scheme with angle of attack feedback is proposed to improve the motion performance. Experiments verify the effectiveness of the proposed motion control method.

## Figures and Tables

**Table 1:** Technical specifications of the robotic dolphin prototype.

Item	Characteristic
Mass	3.9 kg
Dimension (L × W × H)	0.65 m × 0.32 m × 0.14 m
Controller	STM32F407 × 1, STM32F103 × 1
Motor	DC motor × 2, servo motor × 5
Sensor	AHRS, pressure sensor
Power supply	29.6 V rechargeable batteries
Communication unit	Wireless (RF200, 433 MHz)

## Data Availability

The data generated during the current study are available from the corresponding author on reasonable request.
